# Universal human papillomavirus typing by whole genome sequencing following target enrichment: evaluation of assay reproducibility and limit of detection

**DOI:** 10.1186/s12864-019-5598-0

**Published:** 2019-03-20

**Authors:** Tengguo Li, Elizabeth R. Unger, Mangalathu S. Rajeevan

**Affiliations:** Division of High-Consequence Pathogens & Pathology, Centers for Disease Control and Prevention, 1600 Clifton Road, Atlanta, GA 30329 USA

**Keywords:** HPV typing, NGS, Target enrichment, Reproducibility, LOD

## Abstract

**Background:**

We recently described a method for unbiased detection of all known human papillomaviruses (HPV) types with the potential for the determination of their variant and integration from the resulting whole genome sequence data. Considering the complex workflow for target-enriched next generation sequencing (NGS), we focused on the reproducibility and limit of detection (LOD) of this new universal HPV typing assay in this study.

**Results:**

We evaluated the reproducibility and LOD for HPV genotyping based on our recently published method that used RNA-baits targeting whole genomes of 191 HPV types, Agilent SureSelect protocol for target enrichment and Illumina HiSeq 2500 for sequencing (eWGS, enriched whole genome sequencing). Two libraries, prepared from pooled plasmids representing 9 vaccine HPV types at varying input (1–625 copies/reaction), were sequenced twice giving four replicates for evaluating reproducibility and LOD. eWGS showed high correlation in the number of reads mapped to HPV reference genomes between the two flow-cell lanes within (R^2^ = 1) and between experiments (R^2^ = 0.99). The number of mapped reads was positively correlated to copy number (β = 13.9, *p* < 0.0001). The limit of blank (LOB) could be calculated based on mapped reads to HPV types not included in each sample. HPV genotyping was reproducible for all 9 types at 625 copies using multiple cut-off criteria but LOD was 25 copies based on number of reads above LOB even when multiple types were present. eWGS showed no bias for HPV genotyping under single or multiple infection (*p* = 0.16–0.99).

**Conclusions:**

The universal eWGS method for HPV genotyping has sensitivity, competitive with widely used consensus PCR methods with reduced type competition, and with the potential for determination of variant and integration status. The protocol used in this study, using defined samples varying in complexity and copy number, analyzed in replicate and duplicate assays, is applicable to most WGS methods.

**Electronic supplementary material:**

The online version of this article (10.1186/s12864-019-5598-0) contains supplementary material, which is available to authorized users.

## Background

Human Papillomaviruses (HPV) are a group of double-stranded DNA viruses that can cause genital warts and are in the causal pathway for anogenital and oropharyngeal cancers [1]. There are more than 200 HPV types in 5 of the 45 genera within *Papillomaviridae* (*Alphapapillomavirus, Betapapillomavirus, Gammapapillomavirus*, *Mupapillomavirus* and *Nupapillomavirus*). Most assays focus on detection and typing of a subset of *Alphapapillomavirus* HPV types recognized to be clinically important. Conventional typing assays do not capture information on additional viral characteristics, such as integration and variant status within HPV type, therefore, systematic evaluation of the clinical importance of this information is lacking. While next generation sequencing (NGS) could address this limitation, current HPV NGS methods rely on sequencing PCR amplicons targeting a limited region of the genome for genotyping [2–7] or for identifying the integration/variant status [8–16] of a restricted number of types. To overcome these limitations, we recently developed a target enriched whole genome sequencing method (eWGS) designed to identify all known and potentially novel HPV types in a given sample [17]. The resulting whole genome sequence data is useful to address variant and integration status.

Our original eWGS report provided details on the method [target enrichment with RNA baits (based on Agilent SureSelect technology), library preparation, and sequencing using Illumina HiSeq 2500 platform] as well as initial performance metrics for HPV type determination such as genome coverage and uniformity, but reproducibility and limit of detection (LOD) were not addressed. Given the complexity of the workflow for NGS methods, determination of these important characteristics is difficult. A few amplicon-based HPV NGS methods have reported reproducibility in terms of concordance at the level of final type determination; however, these studies did not give detailed measures of variability at the level of base quality, coverage, and mapped reads [2, 5, 6]. Moreover, methods evaluating reproducibility and sensitivity of amplicon-based NGS methods targeting a limited region of the genome are not directly applicable to our conceptually different whole-genome enriched NGS method. This study addresses reproducibility and LOD for type determination based on results in defined HPV samples using our eWGS method. We used overall quality of sequencing reads, number of reads mapped to reference genomes, average depth of coverage, and fraction of genome covered by mapped reads to measure reproducibility and results on samples with decreasing copy number to determine LOD. We find that our eWGS method is highly reproducible for HPV type determination with an LOD of 25 copies/reaction even under the scenario of infection with multiple types.

## Methods

### Samples

Two cell lines known to include HPV 16 (SiHa: 1–2 copies/cell) and HPV 18 (HeLa: ~ 50 copies/cell) were obtained from American Type Culture Collection [ATCC] (Manassas, VA). Cells were cultured according to the recommendations of ATCC. DNA was extracted from the cell pellets collected from cultures in late log phase using DNA isolation kit for cells and tissues (Roche Life Science, Indianapolis, IN). Human placental DNA was obtained from Sigma-Aldrich Corporation (St. Louis, MO). HPV 16 and HPV 18 whole genome plasmid DNA standards (10,000 copies/sample in human placental DNA 100 ng/50 μL TE buffer [10 mM Tris-HCl and 1 mM EDTA (ethylenediaminetetraacetic acid]) were obtained from residual material in an HPV proficiency panel. Plasmids containing the full-length genomes of 9 vaccine HPV types (HPV6, 11, 16, 18, 31, 33, 45, 52, and 58) were received from various sources including ATCC, German Cancer Research Institute (Heidelberg, Germany), Karolinska Institute (Stockholm, Sweden), and Institute Pasteur (Paris, France). Each plasmid clone was expanded in bacterial culture and plasmid DNAs were extracted and purified using Zymo maxiprep kit (Zymo Research, Irvine, CA) to form a working plasmid stock for each HPV type. The HPV type in each plasmid was verified by pattern of restriction enzyme digestion and/or genotype calling by Roche Linear Assay (Roche Diagnostics, Indianapolis, IN). DNA was quantified by Fluorescence-based Qubit dsDNA HS assay on a Qubit 3.0 Fluorometer (Life Technologies, Eugene, OR). HPV genome equivalents (copy number) was calculated based on DNA content.

### Library preparation and sequencing

The bait design, library preparation, HPV enrichment, and deep sequencing followed methods in original publication [17]. Briefly, the custom RNA bait (Agilent Technologies Inc., Santa Clara, CA) included 23, 941 probes (each 120 bases in length) complementary to one strand of the full-length genomes of 191 HPV genotypes/subtypes and 12 probes complementary to *human haemoglobin subunit beta* (*HBB*). Individual libraries with indexing for sample identification were prepared for each sample. Following indexing, equal amounts of 16 libraries were pooled for enrichment by overnight hybridization to the RNA custom bait and the captured fragments were amplified using 14 PCR cycles. The quality and quantity of HPV-enriched, pooled libraries were assessed by Bioanalyzer 2100 (Agilent Technologies, Inc.) and quantitative PCR using KAPA DNA library quantification kit (KAPA Biosystems, Wilmington, MA) on a LightCycler 480 (Roche Diagnostics, Indianapolis, IN). Each pooled library was paired-end sequenced on an Illumina HiSeq 2500 using TruSeq Rapid SBS Kit HS (200 cycle) (Illumina, San Diego, CA).

### Bioinformatics and data analysis

Procedures followed for read de-multiplexing, quality assessment, alignment to *HBB* or HPV reference genomes and cut-off criteria for HPV type determination were as described earlier [17]. Briefly, raw sequence data were de-multiplexed, and the adaptors and barcodes were removed using Illumina BCl2fastq V1.8.4, and reads with base quality Q score were exported as fastq files for batch mapping to HPV and *HBB* reference sequences using CLC genomics workbench 7.5 (CLCbio, Waltham, MA). For this analysis, only reads with no mismatches in the index sequence were used and reference mapping was done by fixing the read length (L) and similarity score (S) at their most stringent level (L1S1).

### Overview of study design

**Fig. 1:**
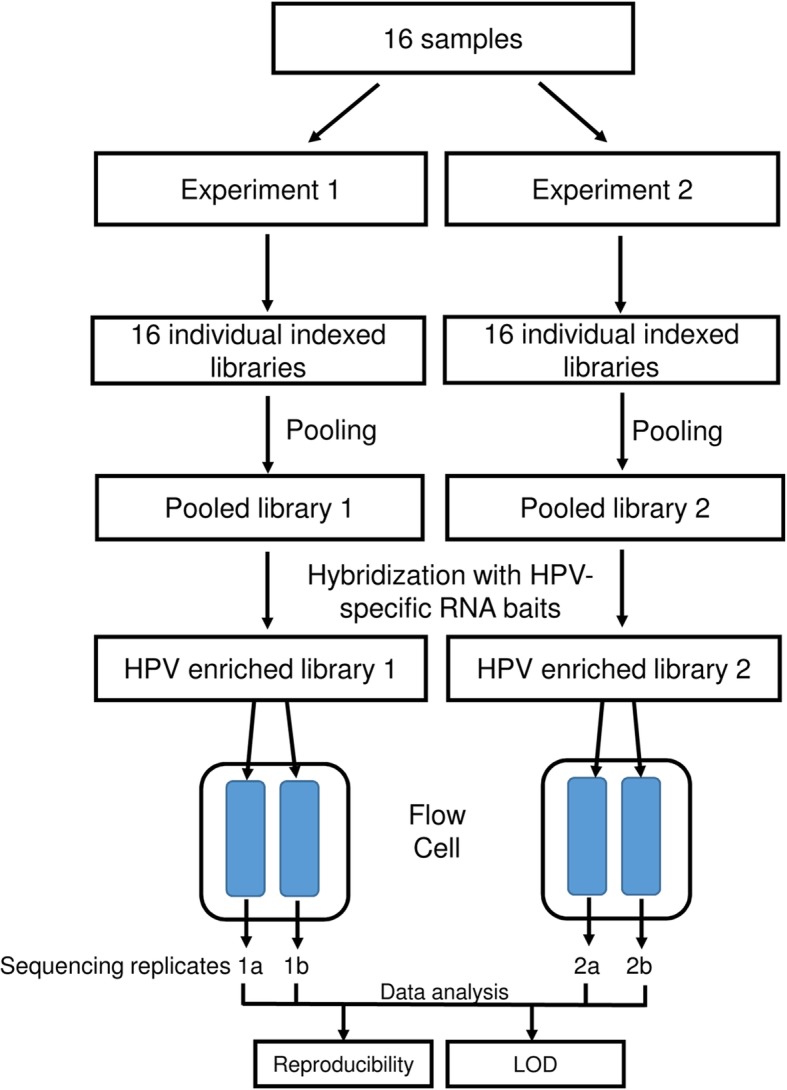
Experimental design for evaluation of reproducibility and limit of detection (LOD) of eWGS. To evaluate reproducibility of results, individual indexed libraries were prepared from 16 samples of defined HPV composition on two occasions 10 days apart (Experiments 1 and 2 in Fig. 1) resulting in 2 pooled libraries. Each library was enriched through hybridization with HPV RNA bait and each enriched library was sequenced on two flow cells. Thus 4 replicate results were obtained for each sample, encompassing experimental replicates (reproducibility of producing enriched library) and sequencing replicates (1a, 1b and 2a, 2b). As each defined sample was a pool of 4 to 5 HPV types with copy number ranging from 625 to 1 (composition shown in Table 1), the limit of detection could be assessed from the replicate results

The study was designed to evaluate the LOD, reproducibility of enriched library preparation as well as the reproducibility of sequencing and identification of HPV types. We used defined samples with known copies of HPV types to evaluate these parameters under controlled conditions. We focused on the 9 HPV types included in the HPV vaccine currently used in the US and prepared mixtures of types to simulate infection with multiple types. We prepared one pool with plasmids for 5 of these types (HPV11, 16, 31, 45, and 52) and the second with plasmids for the four remaining types (HPV 6, 33, 18, and 58). Within each pool, the individual types were present at the same copy number. Each pooled sample was used to make 5-fold serial dilutions (625 copies to 1 copy) in human placental DNA (100 ng/50 μL TE buffer). Serially diluting defined samples of known concentration has been used in previous reports of HPV detection using NGS or PCR or hybrid capture based assays [2, 5, 18, 19], and in World Health Organization’s (WHO) proficiency study of HPV genotyping [20]. The first three columns of Table 1 shows the composition for each of the 16 samples used in this study. The water control and the two cell line DNA (10 ng) samples were prepared without placental DNA. The same 16 samples were used in two experiments evaluating reproducibility and LOD, shown schematically in Fig. 1. In experiment 1, each sample was indexed and combined in equal amounts to form a pooled library, which was then enriched and divided into two replicates for sequencing. Experiment 2 followed the same sample processing, but was performed independently, 10 days after experiment 1.

**Table 1:** Reproducibility of mapping parameters for HPV type determination based on eWGS

Sample No.	HPV types (Expected)	HPV (copy number)	HPV type (eWGS call)	Mapped reads (Mean ± SD)^a^	CV^b^	Average depth (Mean ± SD)	CV	Fraction of genome covered (Mean ± SD)	CV
1	11, 16, 31, 45, 52	625	11	7824.75 ± 1033.9	13.2	98.7 ± 13	13.2	1 ± 0.0009	0.1
			16	6916 ± 1056.3	15.3	87.5 ± 13.4	15.3	0.94 ± 0.017	1.8
			31	6606.75 ± 1099	16.6	83.5 ± 13.9	16.6	1 ± 0.0011	0.1
			45	10,152 ± 1195.1	11.8	129 ± 15.2	11.8	0.99 ± 0.0002	0.0
			52	9860.75 ± 1094.3	11.1	124.2 ± 13.8	11.1	1 ± 0.0008	0.1
2	11, 16, 31, 45, 52	125	11	1858.25 ± 99.2	5.3	23.4 ± 1.3	5.3	0.98 ± 0.0122	1.2
			16	1612 ± 215.9	13.4	20.4 ± 2.7	13.4	0.88 ± 0.0076	0.9
			31	1540.25 ± 49.3	3.2	19.5 ± 0.6	3.2	0.96 ± 0.0085	0.9
			45	2542 ± 84.9	3.3	32.3 ± 1.1	3.3	0.98 ± 0.0038	0.4
			52	2170.5 ± 209.3	9.6	27.3 ± 2.6	9.6	0.99 ± 0.0056	0.6
3	11, 16, 31, 45, 52	25	11	254 ± 67.7	26.6	3.2 ± 0.9	26.6	0.47 ± 0.0879	18.6
			16	264.5 ± 84.3	31.9	3.3 ± 1.1	31.9	0.49 ± 0.0536	11.0
			31	295.25 ± 91.8	31.1	3.7 ± 1.2	31.1	0.59 ± 0.1011	17.1
			45	496.5 ± 56.7	11.4	6.3 ± 0.7	11.4	0.75 ± 0.0245	3.3
			52	351.5 ± 116.6	33.2	4.4 ± 1.5	33.2	0.62 ± 0.0507	8.2
4	11, 16, 31, 45, 52	5	11	91.5 ± 25.8	28.2	1.2 ± 0.3	28.2	0.18 ± 0.0674	37.3
			16	89 ± 47	52.8	1.1 ± 0.6	52.8	0.26 ± 0.1169	45.2
			31	84.5 ± 35.2	41.7	1.1 ± 0.4	41.7	0.16 ± 0.0793	48.4
			45	191.5 ± 67.1	35.0	2.4 ± 0.9	35.0	0.4 ± 0.1125	27.8
			52	58.5 ± 31.1	53.2	0.7 ± 0.4	53.3	0.18 ± 0.0779	43.5
5	11, 16, 31, 45, 52	1	11	12 ± 4.3	36.0	0.2 ± 0.1	36.0	0.05 ± 0.015	28.9
			16	22.75 ± 16.2	71.0	0.3 ± 0.2	71.0	0.11 ± 0.0748	66.8
			31	21.5 ± 5.6	25.9	0.3 ± 0.1	25.9	0.08 ± 0.0289	36.8
			45	38.25 ± 18.3	47.8	0.5 ± 0.2	47.8	0.1 ± 0.0434	42.9
			52	0 ± 0		0 ± 0		0 ± 0	
6	6, 18, 33, 58	625	6	5601.5 ± 141	2.5	70.1 ± 1.8	2.5	1 ± 0.0019	0.2
			18	10,845 ± 1721	15.9	138 ± 21.9	15.9	1 ± 0.0001	0.0
			33	9550.25 ± 893.6	9.4	120.8 ± 11.3	9.4	0.99 ± 0.0007	0.1
			58	10,873 ± 619	5.7	139 ± 7.9	5.7	1 ± 0.0001	0.0
7	6, 18, 33, 58	125	6	1023.75 ± 243.6	23.8	12.8 ± 3	23.8	0.85 ± 0.0103	1.2
			18	2277.25 ± 144.2	6.3	29 ± 1.8	6.3	0.98 ± 0.0049	0.5
			33	1875 ± 62	3.3	23.7 ± 0.8	3.3	0.96 ± 0.0072	0.7
			58	1818.25 ± 212.8	11.7	23.2 ± 2.7	11.7	0.98 ± 0.0082	0.8
8	6, 18, 33, 58	25	6	205.75 ± 17.4	8.5	2.6 ± 0.2	8.5	0.4 ± 0.0625	15.5
			18	258.25 ± 37.5	14.5	3.3 ± 0.5	14.5	0.55 ± 0.0781	14.3
			33	329.25 ± 110.2	33.5	4.2 ± 1.4	33.5	0.54 ± 0.2009	36.9
			58	443.5 ± 56.9	12.8	5.7 ± 0.7	12.8	0.66 ± 0.0429	6.5
9	6, 18, 33, 58	5	6	16.75 ± 11.5	68.5	0.2 ± 0.1	68.5	0.07 ± 0.0443	59.2
			18	60.75 ± 34.8	57.3	0.8 ± 0.4	57.3	0.25 ± 0.088	35.6
			33	70 ± 8.3	11.8	0.9 ± 0.1	11.8	0.24 ± 0.0235	9.8
			58	97.75 ± 41.6	42.6	1.2 ± 0.5	42.6	0.3 ± 0.0832	27.5
10	6, 18, 33, 58	1	6	14.5 ± 16.9	116.7	0.2 ± 0.2	116.7	0.04 ± 0.0452	117.0
			18	9.5 ± 11	116.1	0.1 ± 0.1	116.1	0.08 ± 0.068	84.9
			33	15.75 ± 17.1	108.3	0.2 ± 0.2	108.3	0.05 ± 0.0451	89.0
			58	29.25 ± 4.3	14.9	0.4 ± 0.1	14.9	0.09 ± 0.0251	29.1
11	WHO HPV16	10,000	16	136,765.25 ± 13,297.7	9.7	1729.9 ± 168.2	9.7	0.99 ± 0.0001	0.0
12	WHO HPV18	10,000	18	57,105.5 ± 7675.3	13.4	726.8 ± 97.7	13.4	1 ± 0.0001	0.0
13	H2O		Neg						
14	Placental DNA		Neg						
15	SiHa		16	266,625.5 ± 16,411.4	6.2	3372.4 ± 207.6	6.2	0.92 ± 0.0095	1.0
16	Hela		18	326,684.25 ± 10,950	3.4	4157.9 ± 139.4	3.4	0.63 ± 0.008	1.3

^a^Mean and SD were calculated based on 4 replicates for each sample;

^b^*CV* Coefficient of Variation

Sequencing results from the four replicates were evaluated for overall quality of sequencing reads and alignment parameters for both HPV and *HBB* (number of reads mapped to reference genomes, average depth of coverage, and fraction of genome covered by mapped reads). The reproducibility in the number of reads mapped to reference genomes between flow cell lanes and between experiments was evaluated using linear regression. Reproducibility of HPV type determination was done at L1S1 mapping stringency along with selected cut-off criteria (number of mapped reads ≥1000, average coverage ≥20 and fraction of genome covered ≥0.5), either individually or combined. Mean, standard deviation (SD), and coefficient of variation (CV) for number of mapped reads, depth of coverage and fraction of genome covered for each HPV type in each sample were used as additional measures of reproducibility.

### Determination of limit of detection (LOD)

The LOD for each of the 9 HPV types was calculated based on the number of mapped reads using the equation: LOD = limit of blank (LOB) + 1.64 SD of the lowest concentration tested (1 copy/sample) [21, 22]. Data from all four replicates of the 16 samples was used to calculate LOB and LOD. LOB, representing signal noise, was defined as mean of blank + 1.64SD of blank where blank is defined as the average number of reads mapped to any HPV type that was not expected (false positive reads). Based on this definition, the average number of reads mapped to any unexpected HPV type was 26.4, calculated from a total of 4757 reads in 180 false positive HPV calls from all 16 samples over the four replicates. The experimental LOD was defined as the lowest input copy number with number of mapped reads greater than calculated LOD.

## Results

### Reproducibility of overall eWGS data quality

Pooled libraries were loaded at 3.7 pM in duplicate onto 2 lanes of a flow cell generating a mean cluster density of 916 K ± 52.3 K/ mm^2^ (CV = 5.71%) and 758 K ± 4.24 K/ mm^2^ (CV 0.56%) in experiments 1 and 2, respectively. Both experiments also generated reproducible cluster density (mean = 837 K ± 111.72 K/ mm^2^; CV = 13.34%). Sequence reads (in fastq format) from the 4 replicates had perfect match in the 8 bp index and passed the default filtering of the Illumina BCL2fastq V1.8.4 software. Prior to reference mapping, we evaluated the quality of the sequence data in terms of the mean number of reads, mean base quality score and percent of bases with quality score ≥ 30 (See Additional file 1: Figure S1). The mean number of reads for each sample with DNA ranged from 9684, 372 to 25,100,399 and was highly reproducible among replicates (CV: mean, 14.7%; range, 3.5–27%; Additional file 1: Figure S1A). The water control generated substantially fewer reads (only 0.01% of the total reads) than samples with DNA, and the number of reads among the water control replicates was highly variable (mean = 28, 847 ± 21, 431; CV = 74%) with only 51% of bases having Q scores greater than 30 (mean 20.8) (Additional file 1: Figure S1B). The mean base quality score for the 15 samples with DNA input was 36.7 (CV: mean, 0.5%; range, 0.43 to 0.67%; Additional file 1: Figure S1B) with an average of 94.7% of bases having a quality score greater than 30 (CV mean, 0.61%; range: 0.52–0.77%) (Additional file 1: Figure S1C).

### Reproducibility of *HBB* detection

Reads were mapped to reference sequences of *HBB* under L1S1 mapping stringency. Additional file 2: Figure S2 shows the mean number of reads mapped to *HBB*, average depth of coverage, and fraction of reference genome covered by mapped reads. No reads were mapped to *HBB* in the water control. The mean number of reads mapped to *HBB* in the 4 replicates for the 15 samples with DNA ranged from 12,155 to 37,414 (mean = 25,596) and was highly reproducible among replicates within a sample (mean CV 8.4%; range 3.3–18.8%; Additional file 2: Figure S2A). Similarly, the average depth of coverage (mean CV 8.4%; range 3.3–18.8%; Additional file 2: Figure S2B) and the average fraction of the *HBB* target region covered by mapped reads (mean CV 0.13%; range 0.03–1.36%; Additional file 2: Figure S2C) were highly reproducible among the replicates.

### Reproducibility of HPV detection

**Fig. 2:**
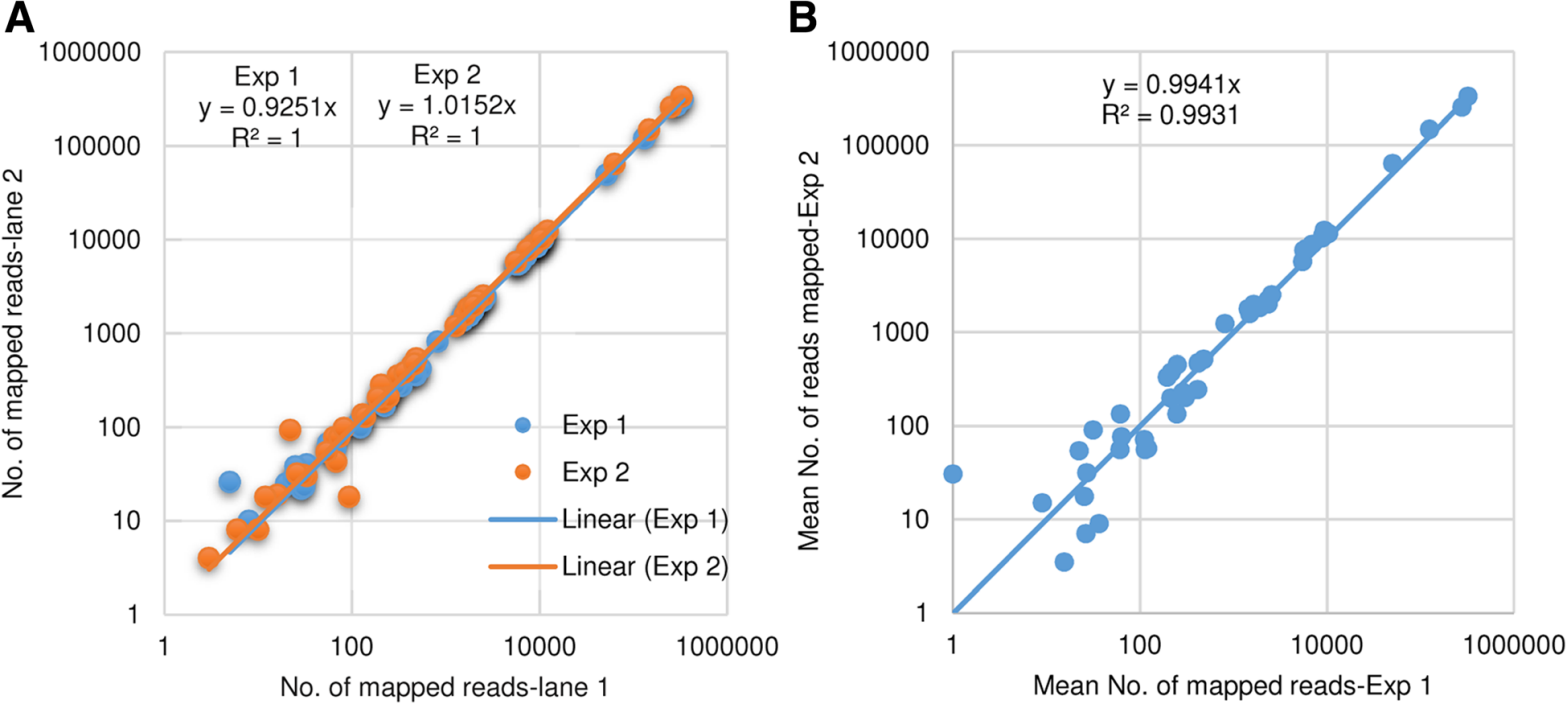
Evaluation of reproducibility in terms of number of reads mapped to expected HPV genomes. **a** reproducibility between 2 flow cell lanes for experiment 1 and experiment 2, and (**b**) reproducibility between experiment 1 and 2 in term of number of mapped reads (mean value of 2 replicates)

Reads were aligned to 191 HPV reference genomes at L1S1 mapping stringency. There was a high correlation in number of reads mapped to HPV genomes between the two flow cell lanes (R^2^ = 1) in both experiments (Fig. 2a) and between the two experiments (R^2^ = 0.99; Fig. 2b). Table 1 shows the mean, SD, and CV (based on 4 replicates) for the number of reads mapped to a specific HPV genome, average depth of coverage, and fraction of reference genome covered. The results for the positive controls (HPV16 and 18 standards, SiHa [HPV16+], and Hela [HPV18+]) were highly reproducible as measured in number of HPV-specific reads mapped (CV range, 3.4 to 13.4%), average depth of coverage (CV range, 3.4 to 13.4%), and fraction of reference genome covered (CV range, 0 to 1.3%).

**Fig. 3:**
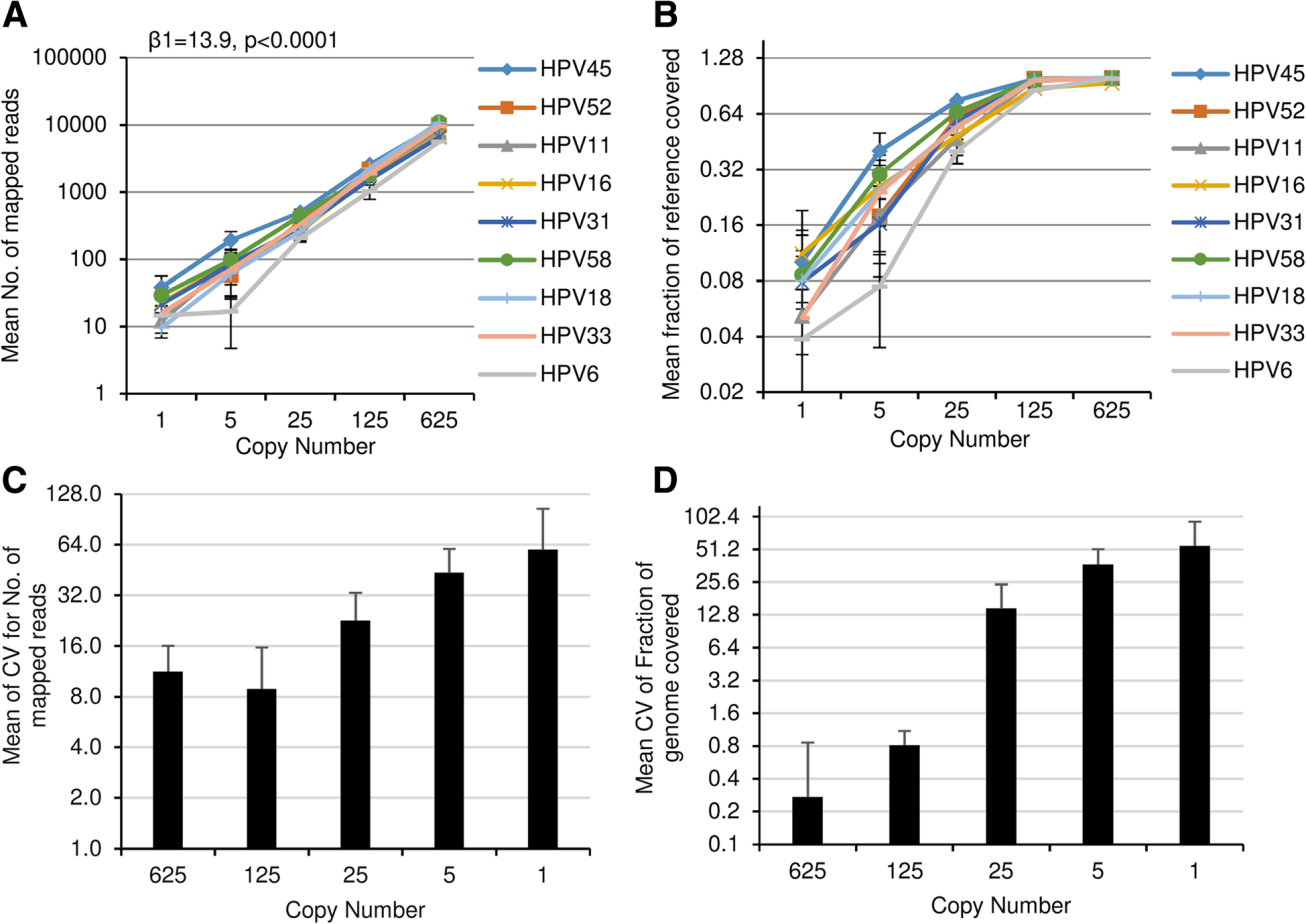
Mapped reads to expected HPV genomes showing the relationship between copy number and (**a**) mean number of mapped reads, **b** fraction of reference genome, and (**c**) mean CV for the 9 HPV plasmids in the number of mapped reads in relation to copy number, and (**d**) mean CV for the HPV plasmids for the fraction of reference genome covered in relation to HPV copy number. Error bars indicates standard deviation

The number of reads mapped to specific HPV genomes in the plasmid pool was correlated to copy numbers, with the number of mapped reads decreasing linearly (β = 13.9, *p* < 0.0001) with decreasing copy numbers (Table 1, Fig. 3a). The fraction of reference genome covered also decreased with decreased copy number, most notably between 125 copies to 1 copy (Fig. 3b). While reproducibility was high for the 9 HPV plasmids at 625 and 125 copies/reaction in terms of the number of mapped reads (mean CV: 10.08%; range: 2.5–23.8%) and fraction of reference covered (mean CV: 0.54%; range: 0–1.8%), it was lower for samples with lower copy numbers (Fig. 3c and d).

We evaluated the reproducibility of type-specific HPV detection in each of the 16 samples in the 4 replicate data sets using each cut-off criterion (number of mapped reads ≥1000, average coverage ≥20 and fraction of genome covered ≥0.5) individually or combined (Table 2). As expected, both negative controls (water and placental DNA) and four positive controls were HPV negative and positive, respectively, with each of the HPV type determination criteria when applied individually or combined. All 4 replicates with 625 and 125 copies/reaction of the 9 HPV type plasmid pool met the mapped reads criterion (> 1000 mapped to the respective genomes) for all 9 types. For the depth of coverage (≥20) and fraction of genome mapped criteria (≥0.5), all replicates of the 9 HPV plasmid pool at 625 and 125 copies/reaction detected all types with the exception that at 125 copies/reaction depth of coverage was not met for HPV types 6, 16 and 31. Therefore, when all three criteria were combined, the replicates reproducibly detected all 9 HPV types in the plasmid pool with 625 copies/reaction but reproducible detection dropped to 6 of 9 types among the 4 replicates at 125 copies/reaction (Table 2). While HPV detection was not reproducible at copy numbers of 25 and below, it should be noted that when considering only the number of reads greater than 1 read or 5 reads used as cut-off in prior reports by Arroyo et al. and Militello et al. respectively [2, 5], then mapped reads specific to all 9 HPV types were detected in all 4 replicates at as low as 5 copies/reaction, simulating multiple infection (Table 1).

**Table 2:** Reproducibility of HPV type determination based on selected eWGS cut-offs^a^

Copy No^c^.	Expected HPV	Concordance^b^			
		No. of reads ≥1000	Depth of coverage ≥20	Fraction of genome covered ≥0.5	All 3 cut-offs combined
625	6	4/4	4/4	4/4	4/4
	11	4/4	4/4	4/4	4/4
	16	4/4	4/4	4/4	4/4
	18	4/4	4/4	4/4	4/4
	31	4/4	4/4	4/4	4/4
	33	4/4	4/4	4/4	4/4
	45	4/4	4/4	4/4	4/4
	52	4/4	4/4	4/4	4/4
	58	4/4	4/4	4/4	4/4
125	6	2/4	0/4	4/4	0/4
	11	4/4	4/4	4/4	4/4
	16	4/4	2/4	4/4	2/4
	18	4/4	4/4	4/4	4/4
	31	4/4	2/4	4/4	2/4
	33	4/4	4/4	4/4	4/4
	45	4/4	4/4	4/4	4/4
	52	4/4	4/4	4/4	4/4
	58	4/4	4/4	4/4	4/4
25	6	0/4	0/4	0/4	0/4
	11	0/4	0/4	2/4	0/4
	16	0/4	0/4	1/4	0/4
	18	0/4	0/4	3/4	0/4
	31	0/4	0/4	4/4	0/4
	33	0/4	0/4	2/4	0/4
	45	0/4	0/4	4/4	0/4
	52	0/4	0/4	4/4	0/4
	58	0/4	0/4	4/4	0/4

^a^Selected eWGS cut-offs are number of mapped reads ≥1000, average depth of coverage ≥20, and fraction of reference genome covered ≥0.5

^b^Level of agreement between eWGS call and expected HPV type in plasmid pool over 4 replicates (4/4 indicates 100% concordance)

^c^Data not shown for 5 and 1 HPV copy/reaction since eWGS did not meet any of the cut-off criteria for type determination

### Evaluation of eWGS for detection bias in multiple infections

To evaluate the potential detection bias among HPV types in multiple infections, we compared the number of mapped reads to all 9 HPV types generated from libraries prepared in plasmid pools as described earlier (simulating multiple infection) with the number of reads generated from libraries prepared with individual plasmids of the same HPV type (simulating single infection). This comparison of single and multiple infections was done with 625 copies of each HPV genome. eWGS showed no bias for detection of all 9 HPV types under single or multiple infection (*p* = 0.16 to 0.99) except for the marginal difference in the number of mapped reads for HPV type 58 (*p* = 0.042) (Table 3).

**Table 3:** Comparison of mean number of mapped reads in samples with multiple and single HPV plasmids

HPV	Pool^a^ (Mean ± SD^b^)	Single (Mean ± SD)	*p*-value
HPV11	9313 ± 597	9571 ± 468	0.68
HPV16	8796 ± 313	7797 ± 626	0.22
HPV31	9010 ± 651	9832 ± 501	0.292
HPV45	14,322.5 ± 1060	16,401 ± 855	0.164
HPV52	11,272 ± 967	12,393 ± 1119	0.400
HPV6	7097 ± 311	7011 ± 378	0.827
HPV18	12,566 ± 874	12,555 ± 943	0.990
HPV33	12,231 ± 859	10,699 ± 726	0.194
HPV58	12,617 ± 991	8509 ± 725	0.042

^a^Pool 1 included HPV 11, 16, 31, 45, and 52; pool 2 included HPV 6, 18, 33, and 58

^b^Mean and SD were calculated based on 2 replicates for each sample

### Determination of limit of detection

The mean number of non-specific HPV reads corresponding to blank resulting from all samples of the 4 replicates was 26.4 ± 65.2, giving rise to a calculated LOB = 133.3 for each of the 9 specific HPV types in the plasmid pool. Based on this LOB, LODs were calculated in terms of number of mapped reads, and the corresponding LOD in terms of copy number was determined for each of the 9 HPV types. LOD for all 9 types in this study was determined to be 25 copies/reaction since each specific HPV type at 25 copies reproducibly generated specific mapped reads greater (mean, 322.1 ± 95.1; range, 205.8–496.5) than calculated LOD (mean, 150.4 ± 10.8; range, 133.3–163.3) in all 4 replicates of the data set (Table 4).

**Table 4:** Limit of detection of eWGS assay for different HPV types

HPV	LOD (No. of mapped reads)	LOD (copy number)	No. of mapped reads at 25 copies of HPV genome (mean ± SD)	Concordnace^a^
HPV11	140.4	25	254 ± 67.7	4/4
HPV16	159.8	25	264.5 ± 84.3	4/4
HPV31	142.4	25	295.3 ± 91.8	4/4
HPV45	163.3	25	496.5 ± 56.7	4/4
HPV52	133.3	25	351.5 ± 116.6	4/4
HPV6	161.1	25	205 ± 17.4	4/4
HPV18	151.4	25	258.3 ± 37.5	4/4
HPV33	161.3	25	329.3 ± 110.2	4/4
HPV58	140.4	25	443.5 ± 56.9	4/4

^a^Concordance indicates the reproducibility of LOD at 25 copies/HPV genome among the 4 replicates

## Discussion

This study provides an in-depth evaluation of the reproducibility and LOD for HPV genotyping with our recently described eWGS method. The approach, using defined samples varying in complexity and copy number, analyzed in replicate and duplicate assays, is applicable to most NGS methods. Importantly, the study design considered reproducibility of the enrichment and library preparation steps as well as sequencing. We chose to use reproducibility of HPV genotyping using reads mapped under the most stringent L1S1 mapping conditions, while varying cut-off criteria for number of reads, depth of coverage, and fraction of reference genome covered. Varying input amounts of HPV genomes (1–625 copies/reaction) and the number of types in the samples provided additional insights into assay robustness and LOD.

eWGS showed high reproducibility in cluster generation between flow cell lanes (CV: 5.71 and 0.56% for experiment 1 and 2, respectively) and between experiments (CV: 13.4%). Consistency of cluster generation reflects consistency during the complex workflow of library preparation and DNA determination of recovered products added to the flow cell. The eWGS results from the 4 replicates demonstrates consistency in the number and quality of reads (mean CV: 14.7% for total number of reads; 0.5% for base quality score; 0.61% for bases with Q score ≥ 30). The number of reads mapped to HPV genomes were highly correlated between the two flow-cell lanes (R^2^ = 1) in both experiments and between the two experiments (R^2^ = 0.99). Importantly, eWGS results in terms of number of mapped reads (mean CV: 10.1%; range: 2.5–23.8%) and fraction of reference covered (mean CV: 0.5%; range: 0–1.8%) at copies/reaction ranging from 125 to 625 was consistent for all 9 HPV plasmids. Using all three cut-off criteria, HPV genotype calling was fully reproducible at 625 copies for all 9 HPV plasmids, while at 125 copies only 6 out of 9 types were called reproducibly among the 4 replicates. HPV 6, 16 and 31 did not meet the depth of coverage criteria ≥20 at 125 copies in all replicates. These results, taken together, suggest that despite the complex nature of its workflow eWGS is robust and highly reproducible for HPV genotyping on a whole genome level at 625 copies/reaction using all three cut-off criteria, with no significant type-specific differences.

As expected, we observed a positive correlation between the number of mapped reads and copy number/reaction (β = 13.9, *p* < 0.0001), as well as the fraction of reference genome covered. Variability among replicates for both measures increased as HPV copy number/reaction decreased. The positive correlation of mapped reads and fraction of genome covered with target concentration as we observed with bait-based eWGS for HPV agrees with a recently reported study on whole genome sequencing of hepatitis C viral genomes following similar target enrichment [23]. However, PCR-based methods either targeting whole genome or amplicon sequencing did not show a copy number dependence with the number of mapped reads [2, 23]. We found that eWGS method for HPV genotyping outperformed an NGS method based on amplicon sequencing [2] with less variability among the 9 vaccine types in the pooled sample for the number of mapped reads at 25 copies/reaction (CV: eWGS, 29.6%; amplicon sequencing, 125.3%). Even at higher input (50–500 copies/reaction) of the 9 vaccine types in the pool, variability in the number of HPV reads with amplicon sequencing remained high (CV: 99.1 to 131.0%) [5]. Amplicon sequencing of replicates of clinical samples for HPV detection also showed high variability in the number of reads between libraries prepared to assess reproducibility (CV: mean 81%; range 2–141%) [2].

Because it is common for multiple HPV types to be present in clinical samples, competition among types for PCR amplification in assays relying on consensus primers could result in a bias in detection [24–26]. The eWGS method should minimize detection bias. We evaluated this by comparing the number of reads for each HPV type at 625 copies in mixed type plasmid pools (simulating multiple infections) and single plasmid pools. The numbers of reads did not vary significantly (*p* = 0.16–0.99) on 8 of the 9 plasmids, suggesting eWGS has minimal bias for HPV typing. The data on reproducibility among the replicates (Table 1) with HPV copies down to 25 copies/reaction (CV for number of reads =29.6%) also indicates low bias for HPV typing.

Reported LODs for NGS assays were determined with cut-offs based on target specific reads and did not consider how LOB could be used in determining the LOD. In this study, we used the number of reads mapped to HPV reference genomes as a parameter in relation to the LOB to calculate LOD [21, 22, 27]. Because we used defined samples in this study, reads mapping to HPV types not included in the sample served as a good measure of false positive reads or “noise”. Using this calculation, 25 copies/reaction was determined to be the LOD for the 9 types with eWGS method. At an LOD of 25 copies/reaction, eWGS averaged 322 ± 95.4 mapped reads for HPV genotype calling, higher than the 1–5 mapped reads used as threshold by some amplicon sequencing methods [2, 5]. This LOD compares favorably with PCR methods for HPV detection and typing that have reported sensitivity ranging from 50 to 500 copies/reaction [5, 20] and sensitivity at 100 copies/reaction for another HPV genotyping method using hybrid capture combined with isothermal whole genome amplification and Luminex detection [19]. There are a few studies [23, 28–32] addressing the determination of LOD using NGS methods for viral detection but the units of LOD (genome copies/ml, pfu/ml, IU/ml) reported in these studies are not comparable to genome copy number/reaction used in this study for HPV typing.

Our study is not without limitations, and additional work will be required to evaluate eWGS performance using clinical samples, and for other variables such as inter-operators, instrument, and laboratory variation. Determination of analytical sensitivity of an HPV detection assay is challenging because biologic samples vary widely in the proportion of normal and infected/neoplastic cells. Therefore, the best option for HPV is the determination of sensitivity denoted as copies or genomes/reaction as reported previously for NGS or PCR or hybrid capture based assays [2, 5, 18, 19], and in WHO’s proficiency study of HPV genotyping [20]. Assay performance is likely to vary when applied to standard testing conditions; the challenge of determining true results in undefined samples can only be addressed indirectly using inter-assay comparisons. Cost reductions could be achieved through automating the steps of library production and by increasing the number of samples that are pooled for the sequencing reaction. In our continued work (data not shown), we tested a total of 18 HPV types from mixing 4–7 types/reaction, and found that the number of samples/sequencing lane can be increased from 16 to 32 while maintaining the level of sensitivity (625 copies/reaction) for type determination (based on all three cut-off criteria combined) for all 18 HPV types. Additional work is needed to determine the maximum number of samples that can be pooled per sequencing reaction without compromising sensitivity. The focus of our current study was to verify sensitivity and reproducibility of HPV detection at the level of type. We are developing a bioinformatics pipeline for HPV genotyping that can be modified to identify variant and integration status from the same capture DNA sequencing data. HPV integration may be inferred from identifying single end or paired end reads that map to both the human and viral reference genomes as well as by identifying missing segments of HPV genome, as noted in our first publication [17]. In summary, we report an in-depth evaluation of the reproducibility of eWGS method for HPV genotyping employing a variety of metrics that include overall quality of reads, number of reads mapped, depth of coverage, and fraction of reference genome covered. Results indicate eWGS is highly reproducible for HPV genotyping at 625 copies /reaction using multiple cut-off criteria, with the possibility of reducing LOD to 25 copies/reaction, if HPV detection is based on the widely used number of mapped read criteria alone.

## Conclusions

Our data suggest that eWGS method reduces type-competition, and has sensitivity competitive with widely used consensus PCR methods for HPV genotyping. In addition to genotyping, eWGS has the potential to provide highly reproducible and less biased sequence data for variant determination and identifying integration status, but may require additional studies to determine LODs specific to these applications of eWGS. The protocol used in this study, involving defined samples varying in complexity and copy number, analyzed in replicate and duplicate assays, is applicable to most WGS methods.

## Additional files

Additional file 1: **Figure S1.** Overall quality of reads from 4 replicates. (A) Mean number of reads passing the default filtering of Illumina BCL2fasq V1.8.4. (B) Mean base quality score. (C) Percentage of base with quality score ≥ 30. Y-axis indicates the mean of values of 4 replicates. Error bars represent standard deviation of 4 replicates. (PDF 107 kb)

Additional file 2: **Figure S2.** Reproducibility for eWGS for internal control *HBB*. Data shown is mean ± SD of 4 replicates/sample for number of reads (A), average depth of coverage (B), and fraction of reference covered (C). Error bars represent standard deviations. (PDF 94 kb)

## Abbreviations

ATCC: American type culture collection
CV: Coefficient of variation
EDTA: Ethylenediaminetetraacetic acid
eWGS: enriched whole genome sequencing
*HBB*: *Human haemoglobin subunit beta*
HPV: Human papillomavirus
LOB: Limit of blank
LOD: Limit of detection
NGS: Next generation sequencing
SD: Standard deviation
TE buffer: Tris-EDTA buffer
WHO: World Health Organization
