# Supplementary material for: Universal human papillomavirus typing by whole genome sequencing following target enrichment: evaluation of assay reproducibility and limit of detection
Source: BMC Genomics. 2019 Mar 20;20:231. doi: 10.1186/s12864-019-5598-0 (PMC6425667; doi:10.1186/s12864-019-5598-0)

Additional file 1, Supplementary Figure 1. Overall quality of reads from 4 replicates. (A) Mean number of reads passing the default filtering of Illumina BCL2fasq V1.8.4. (B) Mean base quality score. (C) Percentage of base with quality score ≥ 30. Y-axis indicates the mean of values of 4 replicates. Error bars represent standard deviation of 4 replicates (PDF).

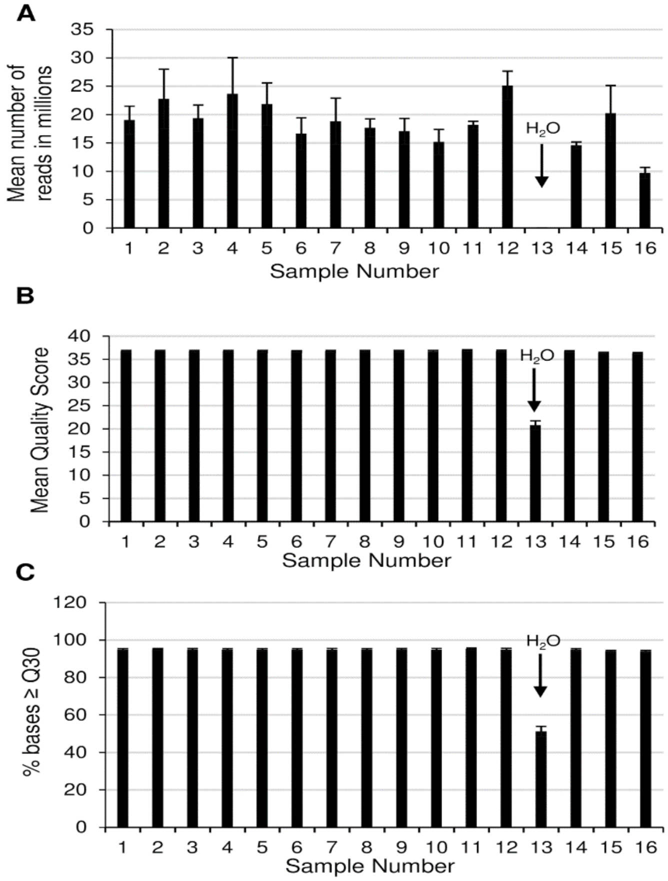

Supplement: Supplementary file 1 — Figure S1. Overall quality of reads from 4 replicates. (A) Mean number of reads passing the default filtering of Illumina BCL2fasq V1.8.4. (B) Mean base quality score. (C) Percentage of base with quality score ≥ 30. Y-axis indicates the mean of values of 4 replicates. Error bars represent standard deviation of 4 replicates. (PDF 107 kb) [file 12864_2019_5598_MOESM1_ESM.pdf]
