# Supplementary material for: Universal human papillomavirus typing by whole genome sequencing following target enrichment: evaluation of assay reproducibility and limit of detection
Source: BMC Genomics. 2019 Mar 20;20:231. doi: 10.1186/s12864-019-5598-0 (PMC6425667; doi:10.1186/s12864-019-5598-0)

Additional file 2, Supplementary Figure 2. Reproducibility for eWGS for internal control *HBB*. Data shown is mean ± SD of 4 replicates/sample for number of reads (A), average depth of coverage (B), and fraction of reference covered (C). Error bars represent standard deviations (PDF).

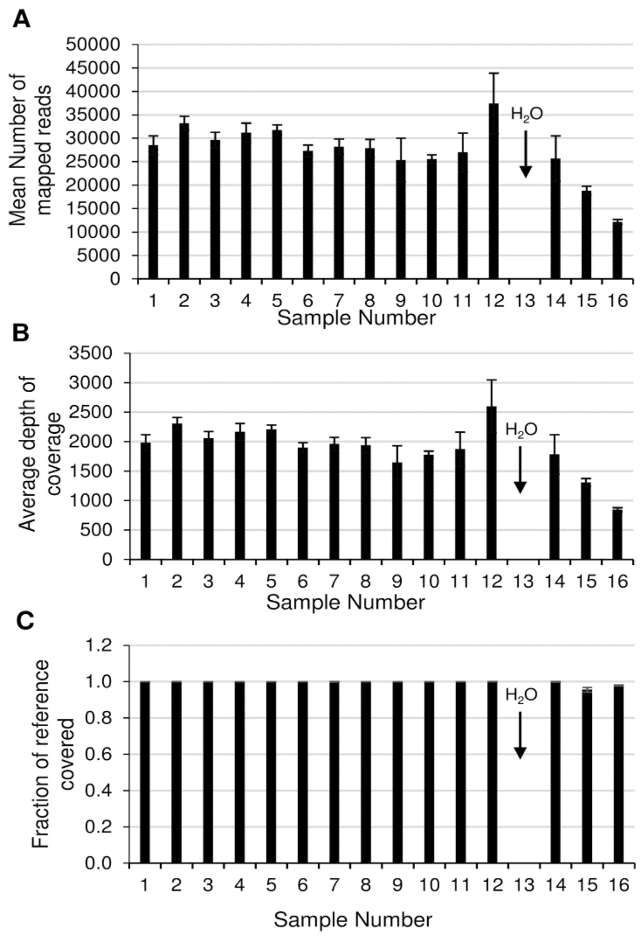

Supplement: Supplementary file 2 — Figure S2. Reproducibility for eWGS for internal control HBB. Data shown is mean ± SD of 4 replicates/sample for number of reads (A), average depth of coverage (B), and fraction of reference covered (C). Error bars represent standard deviations. (PDF 94 kb) [file 12864_2019_5598_MOESM2_ESM.pdf]
